# Biomarkers of Type II Synthetic Pyrethroid Pesticides in Freshwater Fish

**DOI:** 10.1155/2014/928063

**Published:** 2014-04-23

**Authors:** Anilava Kaviraj, Abhik Gupta

**Affiliations:** ^1^Department of Zoology, University of Kalyani, Kalyani, West Bengal 741235, India; ^2^Department of Ecology & Environmental Science, Assam University, Silchar, Assam 788011, India

## Abstract

Type II synthetic pyrethroids contain an alpha-cyano group which renders them more neurotoxic than their noncyano type I counterparts. A wide array of biomarkers have been employed to delineate the toxic responses of freshwater fish to various type II synthetic pyrethroids. These include hematological, enzymatic, cytological, genetic, omic and other types of biomarkers. This review puts together the applications of different biomarkers in freshwater fish species in response to the toxicity of the major type II pyrethroid pesticides and assesses their present status, while speculating on the possible future directions.

## 1. Introduction

Pyrethroids are synthetic derivatives of pyrethrins, the toxic component contained in the flower of the plant* Chrysanthemum cinerariaefolium* [1]. The insecticidal properties of pyrethrins are derived from ketoalcoholic esters of chrysanthemic and pyrethroic acids, which are strongly lipophilic in nature and have the ability to penetrate rapidly into the body of insect pests to produce toxicosis. However, the naturally occurring pyrethrins are extremely sensitive to light and break down in a few hours, before adequate quantity of pyrethrins is accumulated in the body of insects to kill them. Interesting developments were held during the last twenty years in modifying structure, stereochemistry, and formulations to develop thousands of modern pyrethroids with chemical properties and biological performance suitable for agriculture field. These modifications include halogenations of the cyclopropane side chain of the pyrethrin molecule, addition of cyano group in the alcohol moiety [2], mixing of geometric and optical isomers, and formulations of the technical grade (containing 85% or more of the active ingredients) compound with carriers and solvents. The pyrethroids containing alpha-cyano group are grouped as type II and noncyano pyrethroids are grouped as type I pyrethroids (Table 1). Type II (*α*-cyano) pyrethroids are more potent neurotoxicants than type I (noncyano) pyrethroids, with toxicity being solely attributed to *α*-cyano substituent [2, 3].

Synthetic pyrethroid pesticides, especially the type II compounds, are extensively used in pest management all over the world because of their relatively low toxicity to birds and mammals. However, they are known to have pronounced toxic effects on bees, freshwater fish, and other aquatic organisms [4, 5]. It is, therefore, necessary to evaluate the current status of the various biomarkers used for delineating the toxic effects of type II synthetic pyrethroids on freshwater fish and envisage the directions that the biomarker research may take in near future. A biomarker is defined as “a change in biological response which can be related to exposure to or toxic effects of environmental chemicals” [6]. The level of the response could range from those generated at molecular levels to those at the community or even the ecosystem level [6]. Against this broad background, the present review attempts to provide a synthesis of the knowledge base available on the biochemical and physiological biomarkers of the type II pyrethroids in freshwater fish identifying the parameters which can be established as unequivocal biomarkers of these pesticides.

## 2. Hematological Biomarkers

Although somewhat simple and largely nonspecific, various properties of blood and serum have long been used as biomarkers of xenobiotic toxicity, including pesticides, in fish. The most common blood parameters monitored include total erythrocyte count, total leucocyte count, and haemoglobin and haematocrit content, which were found to decrease on cypermethrin exposure in the Indian major carps* Labeo rohita* and* Catla catla* [7]. However, several workers [8–11] recorded increased lymphocyte, leucocyte, and erythrocyte counts, packed cell volume, and hemoglobin in several species of fishes challenged with cypermethrin, deltamethrin, and fenvalerate. Jayaprakash and Shettu [12] observed a decrease in hemoglobin content, total erythrocyte count, packed cell volume, mean corpuscular volume, and mean corpuscular hemoglobin concentration and an increase in total leukocyte count, mean corpuscular volume, erythrocyte sedimentation rate, and clotting time values in the snakehead,* Channa punctatus,* exposed to deltamethrin. Other hematological variables such as total serum protein, albumin, and globulin contents, albumin-globulin ratio, plasma glucose, alanine aminotransferase, and cholinesterase were reduced in fishes exposed to cypermethrin and deltamethrin [7, 13]. On the contrary, significantly higher erythrocyte count, haemoglobin, haematocrit, plasma total protein, albumins, calcium, and ammonia contents, and activities of aspartate aminotransferase and creatine kinase were observed in the deltamethrin-treated group compared to the control group [13]. Thus the hematological biomarkers, though relatively simple to measure and fairly useful in detecting xenobiotic effects, are of rather limited utility and often somewhat contradictory in their response.

## 3. Hyperglycemia as a Biomarker 

Reduction in hepatic glycogen accompanied by a rise in blood glucose is a common reaction of fish against xenobiotic insult. Cypermethrin induced hyperglycaemia has been recorded in Indian major carp* Labeo rohita* [14], Korean rock fish* Sebastes schlegeli* (Hilgendorf) [15], rainbow trout* Oncorhynchus mykiss* [16], and Asian air breathing fish* Heteropneustes fossilis *[17]. Depletion of hepatic glycogen due to cypermethrin treatment has been observed in* Tilapia mossambica* [18] and* Clarias batrachus* [19]. Glycogen depletion is a regulatory step against xenobiotic insult. It increases intermediary metabolism resulting in the protection of the hepatocytes. Increase in the activity of lactate dehydrogenase (LDH), an enzyme responsible for conversion of pyruvate to lactate in fish exposed to sublethal concentration of cypermethrin [16, 20, 21] and *λ*-cyhalothrin [22], is an indication of such metabolism. Hyperglycaemia, though not specific, is a quick response of the adverse effects of the toxic doses of type II pyrethroids.

## 4. Enzymes of Energy Metabolism as Biomarkers

The enzymes of energy metabolism have also been found to have the potential to be used as biomarkers of type II pyrethroid toxicity in fish. Total adenosine triphosphatase, sodium-potassium adenosine triphosphatase, and magnesium adenosine triphosphatase activities in the gills of* Catla catla* were significantly reduced when exposed to sublethal concentrations of cypermethrin [7]. Cypermethrin-challenged* Clarias batrachus* showed significant inhibition of the activities of total, Mg^+2^, and Na^+^-K^+^ ATPase enzymes, while it increased the levels of glycogen phosphorylase (a), thereby suggesting that these enzymes had the potential to be used as biomarkers of cypermethrin exposure in fish [23]. Deltamethrin also inhibited the activities of Na^+^-K^+^ ATPase in the gill and heart tissues of the freshwater fish* Ancistrus multispinis* [5]. Liver alkaline phosphatase is also known to play a role in glycogen metabolism. The enzyme is capable of inactivating phosphorylase enzymes and promotes glycogen synthesis. Inhibition of alkaline phosphatase activity in the liver is thus related to the breakdown of glycogen to meet the energy demand under stress or decrease in the rate of transphosphorylation or uncoupling of oxidative phosphorylation. Saha and Kaviraj [17] observed that alkaline phosphatase activity in the liver of* H. fossilis* was reduced after exposure to 0.3–0.5 *μ*g L^−1^ cypermethrin. Das and Mukherjee [14] also observed a reduction of alkaline phosphatase in the brain tissue of* Labeo rohita* following exposure to cypermethrin. But there are reports that alkaline phosphatase activity of fish increases after exposure to cypermethrin [8, 15, 20, 24]. Higher activities of lactate dehydrogenase (LDH) and acid and alkaline phosphatases were also observed in* Channa punctatus* exposed to cypermethrin and *λ*-cyhalothrin [22]. Necrosis of liver and subsequent leakage of alkaline phosphatase into blood stream might be responsible for an increase of the enzyme in blood [25].

## 5. Oxidative Stress Biomarkers

Lipid peroxidation, induced by reactive oxygen species (ROS), is a common oxidative stress biomarker of toxicants. Lipid peroxides are capable of modifying the properties of biological membranes, eventually resulting in damage of cellular membrane [26–28]. The most widely used assay for lipid peroxidation is malondialdehyde (MDA), a secondary product of lipid peroxidation caused by free radicals [22, 29]. Fish, like other organisms, have evolved defense systems comprising both enzymatic and nonenzymatic processes to minimize cell damage by lipid peroxides. Catalase (CAT), an enzymatic antioxidant, scavenges ROS and converts them to less reactive species thereby preventing lipid peroxidation. Fish exposed to deltamethrin showed a tendency towards increase in the level of MDA and reduction in the activity of CAT, although cadmium pre-exposure at low concentrations appeared to modulate deltamethrin-induced oxidative stress [30, 31].* Channa punctatus* exposed to alphamethrin also showed a decrease in the activity of CAT [32]. Other type II synthetic pyrethroids such as *λ*-cyhalothrin [33], cypermethrin [34, 35], cyfluthrin [36], deltamethrin [37, 38], fenvalerate [39], cypermethrin, and *λ*-cyhalothrin [40] also increased lipid peroxidation and/or reduced CAT activity in different species of freshwater fish. Dinu et al. [41] observed that superoxide dismutase (SOD) and catalase (CAT) activities were reduced in the liver along with activation of glutathione peroxidase (GPX), glutathione transferase (GST), and glutathione reductase (GR), while an increase in CAT, GST, and GR was recorded in the intestine. Since liver is involved in detoxification of xenobiotics, while intestine is engaged in absorption, this difference could be linked to their specific metabolic pathways. This finding, therefore, calls for tissue-specific understanding and application of the biomarker approach, rather than adopting a more generalized one common for all tissues and organisms. However, several studies have also reported increased CAT activity in fish exposed to type II pyrethroids such as cypermethrin [42].

Cytochrome P450 and its numerous isomers comprise a very important set of enzymatic oxidants that are actively involved in the bioelimination of xenobiotics entering the body [43]. Bhutia et al. [44] found that several cytochrome P450 isozymes were induced in the hepatic microsomes of* Heteropneustes fossilis* exposed to cypermethrin, while some isozymes were inhibited when compared to that in the control. The total cytochrome P450 content was significantly induced upon cypermethrin exposure.

Ascorbic acid serves as a nonenzymatic antioxidant biomarker of oxidative stress of pyrethroid pesticides to fish [45]. Saha and Kaviraj [17] observed that ascorbic acid level depleted in liver and kidney of* H. fossilis* in response to 0.5 *μ*g/L of cypermethrin, probably because of involvement of ascorbic acid in counteracting the stress of cypermethrin. When fish were fed ascorbic acid through diet, ascorbic acid level increased in liver and kidney but decreased in blood of cypermethrin-treated fish as compared to control. Liver and kidney probably accumulated ascorbic acid from the blood stream as a defense mechanism against cypermethrin.

## 6. Enzymes of Nitrogen Metabolism as Biomarkers

Increased activities of enzymes of nitrogen metabolism such as aspartate aminotransferase (AST), alanine aminotransferase (ALT), glutamate dehydrogenase, and glutamine synthetase in response to cypermethrin [46] and *λ*-cyhalothrin [40] exposure served as useful biomarkers in fish. Jee et al. [15] found an increase in the levels of serum glutamic-acid oxaloacetic-acid-transaminase and glutamic-acid pyruvic-acid-transaminase in Korean rockfish (*Sebastes schlegeli*) exposed to cypermethrin. Velisek et al. [16] found an increase in the activity of aspartate aminotransferase (AST), creatine kinase (CK), and lactate (LACT) in rainbow trout after exposure to cypermethrin. Increase in the activities of ALT, AST, and transaminases appeared as specific reaction of type II pyrethroids. Alanine aminotransferase (ALT) and aspartate aminotransferase (AST) activities increased in response to cypermethrin in* Orecochromis niloticus* [20]. Marked increase in the activities of transaminases in* Clarias batrachus* [47] and an increase in the activities of aspartate and alanine aminotransferase in* Prochilodus lineatus *have also been reported in response to cypermethrin, a type II pyrethroid [24].

## 7. Acetylcholinesterase (AChE) Activity as Biomarker

AChE activity has been established as a potent biomarker for organophosphorus and carbamate pesticides, while unequivocal evidence is lacking for organochlorines. The activity of the AChE was not inhibited by deltamethrin in three species of fishes [5, 48–50]. Similarly, esfenvalerate exposure had no effect on AChE activity. On the contrary both k-cyhalothrin and cypermethrin exhibited tissue-specific as well as dose dependent decrease in the activity of AChE, with a greater inhibition of AChE activity in brain when compared to that in muscle and gills [51]. Thus the evidence for AChE inhibition by type II pyrethroids is not unequivocal and needs further investigations in different species and a broad range of these pesticides.

## 8. Gene Expression as Biomarker

Esfenvalerate exposure invoked alterations in the expression of genes associated with immune responses, along with apoptosis, redox, osmotic stress, detoxification, and growth and development. Swimming impairment correlated significantly with expression of the enzyme aspartoacylase (ASPA), which is involved in brain cell function. Selected genes were investigated for their use as molecular biomarkers, and strong links were determined between measured downregulation in ASPA and observed behavioral responses in fish exposed to environmentally relevant pyrethroid concentrations. Microarray technology proved to be a useful approach in screening for and generation of molecular biomarkers in endangered, nonmodel organisms. It was especially valuable for identifying specific genes that could be directly linked with sublethal toxicological endpoints such as changes in expression levels of neuromuscular genes resulting in measurable swimming impairments in larval delta smelt [52]. Shi et al. [42] found that cypermethrin downregulated ogg1 and increased p53 gene expression as well as the caspase-3 activity in zebra fish embryos, resulting in oxidative stress and apoptosis. Thus gene expression holds promise as a more specific biomarker of type II pyrethroid toxicity. However, further researches are required to explore its application under different situations and in a wide range of species.

## 9. Genotoxic Effects as Biomarkers

Chromosomal aberrations (CA), nuclear abnormalities (NA), and the micronucleus test (MN) are the most commonly employed measures of genotoxicity. Campana et al. [53] provided one of the early validations of the MN test in fishes for the assessment of genotoxic pollutants, when they studied the genotoxicity of the pyrethroid *λ*-cyhalothrin in erythrocytes of* Cheirodon interruptus interruptus.* The results obtained demonstrated the genotoxic effects of this pyrethroid in the fish. Çavaş and Ergene-Gözükara [54] showed that nuclear (micronucleus frequency) and nucleolar (changes in quantitative characteristics of nucleoli) biomarkers could be gainfully used to evaluate the functional and structural genotoxic effects of the type II synthetic pyrethroid *λ*-cyhalothrin on the fish* Garra rufa*. The frequency of micronucleated erythrocytes was significantly increased in exposed fish while the nucleolar parameters were repressed, thereby demonstrating the genotoxic potential of *λ*-cyhalothrin. These findings also suggested that the use of nucleolar biomarkers on fish fin cells, in addition to micronucleus test, could provide valuable information in aquatic genotoxicity studies. Velmurugan et al. [55] observed that different types of chromosomal aberrations such as chromatid breaks, centromeric fusion, acentric fragments, chromosomal gaps, sticky plates, aneuploidy, and ring chromosomes could serve as useful biomarkers of *λ*-cyhalothrin in the fish* Mystus gulio* exposed to 6.4 ppb of this pyrethroid.

In subsequent studies, it was shown that deltamethrin readily induced MN and NAs along with increased lipid peroxidation [56], while cypermethrin also showed increased frequencies of CA and MN in a concentration-dependent manner [57]. Exposure to *λ*-cyhalothrin induced MN and NAs in the mosquito fish* Gambusia affinis*, with the NAs comprising blebbed, lobed, and notched nuclei. The ratios of polychromatic to normochromatic erythrocytes were also found to decrease in pesticide-challenged mosquito fish [58]. These results suggest that genotoxic damage could be a useful biomarker in fish exposed to type II synthetic pyrethroids.

## 10. Omic Biomarkers

The emerging field of “omics” that comprises genomics, proteomics, metabolomics, and others is expected to have increasing applications in the field of ecotoxicology by establishing newer biomarkers of environmental contaminants [59]. Metabolomics is emerging as a new and robust approach towards the study of xenobiotic insult. The applicability and potential of the metabolomics approach for the elucidation of toxicological effects of pesticides and the underlying mechanisms was examined in a study, which used a ^1^H nuclear magnetic resonance (NMR) based metabolomics approach to investigate the toxicity of *λ*-cyhalothrin in goldfish (*Carassius auratus*). The *λ*-cyhalothrin exposure influenced levels of many metabolites, such as leucine, isoleucine, and valine in brain and kidney; lactate in brain, heart, and kidney; alanine in brain and kidney; choline in brain, heart, and kidney; taurine in brain, heart, and kidney; N-acetylaspartate in brain;* myo*-inositol in brain; phosphocreatine in brain and heart; 2-oxoglutarate in brain;* cis*-aconitate in brain, and broke the balance of neurotransmitters and osmoregulators. It also caused oxidative stress and disturbed metabolisms of energy and amino acids. The implication of glutamate—glutamine—gamma-aminobutyric axis in *λ*-cyhalothrin induced toxicity was demonstrated. Thus the study was a positive step towards the discovery of precise biomarkers for pesticide pollution in aquatic environment [60]. Besides, proteomic approaches have been utilized to assess the effects of zinc, cadmium, and various cyanotoxin exposures in fish [59]. It is expected that similar approaches would be increasingly explored for their adoption and incorporation into the diverse biomarker repertoire for fish.

## 11. Specificity in Sodium Channel Interactions 

Several researchers have attempted to elucidate molecular mechanism of action of type I and II pyrethroids and specificity in their interactions with voltage-sensitive sodium channels [2, 61, 62]. Type II pyrethroids are known to inhibit the deactivation of sodium channels more than that by their type I counterparts. Insect sodium channels are large transmembrane proteins composed of four homologous domains (I to IV). While studying point (amino acid) mutations at various regions of insect sodium channels of pyrethroid-resistant insect population it was observed that the lack of glycine at position 1111 (G^1111^) and charge neutralization or reversal of two positively charged lysines immediately downstream in the intracellular linker connecting domains II and III of the sodium channels of cockroach turned them more resistant to type II but not to type I pyrethroids [61]. G^1111^ and two downstream lysines are thus assumed to be involved selectively in the response of sodium channels to type II pyrethroids due to their direct or indirect interaction with the *α*-cyano group. Interactions of type II pyrethroids with voltage-sensitive sodium channel are also well documented in mammals [2]. But hardly is there any information on pyrethroid-sodium channel interaction in fish and there is a need to concentrate researches in this field for identifying specific biomarkers of type II pyrethroid toxicity.

## 12. Conclusions

Biomarkers of oxidative stress appear to have been the most widely used category of biomarkers of type II synthetic pyrethroids in the last decade. The accentuated generation of ROS due to the introduction of the alpha-cyano group in these pesticides could be a possible reason behind the increased reliance on this type of biomarkers. However, in their quest for more specificity, researchers have also investigated gene expression including involvement of specific amino acids in sodium channel in the recent years, with metabolomics and proteomics forming the frontier area of biomarker research. These trends are being facilitated by the development of increasingly precise and sensitive instrumentation such as microarray and NMR.

## Figures and Tables

**Table 1:** Pyrethrins, type I pyrethroids, and type II pyrethroids*.

Active ingredient	Pyrethrins: constituents of natural pyrethrum extracts		Synthetic derivatives of pyrethrin: pyrethroids	
	Esters of Chrysanthemic acid	Esters of Pyrethric acid	Type I Esters without alpha-cyano group	Type II Esters with alpha-cyano group
Types of pyrethrins/pyrethroids gradually developed	Pyrethrin-I	Pyrethrin-II	Allethrin 1st generation	Fenvalerate 3rd generation
	Cinerin-I	Cinerin-II	Resmethrin 2nd generation	Cyhalothrin 4th generation
	Jasmolin-I	Jasmolin-II	Permethrin 3rd generation	Cypermethrin 4th generation
			Bifenthrin 4th generation	Deltamethrin 4th generation

*Based on [1, 2].
